# The Prognostic Effect of Sarcopenia in Solid Cancers Treated with Immunotherapy: A Systematic Review and Meta-Analysis

**DOI:** 10.3390/jcm15072720

**Published:** 2026-04-03

**Authors:** Laura F. J. Huiskamp, Anouk W. M. A. Schaeffers, Lot A. Devriese, Remco de Bree

**Affiliations:** 1 Department of Head and Neck Surgical Oncology, University Medical Center Utrecht, Heidelberglaan 100, 3584 CX Utrecht, The Netherlands; l.f.j.huiskamp@umcutrecht.nl (L.F.J.H.); a.w.m.a.schaeffers-2@umcutrecht.nl (A.W.M.A.S.); 2 Department of Medical Oncology, University Medical Center Utrecht, Heidelberglaan 100, 3584 CX Utrecht, The Netherlands; l.a.devriese@umcutrecht.nl

**Keywords:** sarcopenia, skeletal muscle mass, solid tumours, prognosis, meta-analysis

## Abstract

**Background:** Sarcopenia, defined as the presence of low skeletal muscle mass, is highly prevalent in cancer patients and associated with adverse events and lower survival. Given the chronic inflammation associated with sarcopenia, it is especially relevant in patients receiving immunotherapy. The current research is limited to small sample sizes and single cancer types leaving the overall effect of sarcopenia unclear. This review and meta-analysis examined the prognostic effect of sarcopenia in cancer patients treated with immunotherapy. **Methods:** A systematic review was conducted using EMBASE, MEDLINE, Cochrane, and Scopus. Studies were included if they investigated the association of baseline low skeletal muscle mass, as measured on CT or MRI and normalised for height, and survival in patients with solid tumours treated with immunotherapy. A meta-analysis of hazard ratios (HR) was performed using a random effects model for overall survival (OS) and progression-free survival (PFS). **Results:** In total, 48 studies were included in this review, of which 42 were included in the meta-analysis. The prevalence of sarcopenia ranged between 19.5% and 83.6%. The meta-analysis showed an overall prognostic effect of sarcopenia for OS and PFS (HR = 1.58; 95% CI 1.35–1.85; *p* < 0.0001 and HR = 1.50; 95% CI 1.30–1.72; *p* < 0.001, respectively). High heterogeneity was present between the included studies, which could possibly be explained by the differences in sarcopenia definition, cutoff values, and measurement techniques. **Conclusions:** Sarcopenia is a significant adverse prognostic factor for both OS and PFS in patients with solid tumours treated with immunotherapy across cancer types. Further research into the underlying mechanism of sarcopenia and its relation to the immune response and survival is needed. Prospective intervention studies are required to establish the care needed, such as nutrition and exercise, to improve the prognosis of sarcopenic patients.

## 1. Introduction

There are an estimated 20 million new cancer cases each year [1], with cancer set to become the number one cause of death in Europe [2]. Over the past decade, immunotherapy has emerged as a promising treatment approach and offers novel treatment options for many patients [1]. In contrast to traditional treatment, immunotherapy harnesses the patient’s immune system to treat cancer with more precision and long-term disease control [1]. Immunotherapy was initially introduced for the treatment of melanoma and lung cancer. In recent years, indications have expanded and several types of immunotherapy have been developed and used in the treatment of many cancer types, such as renal, hepatocellular, urothelial, and head and neck cancer [1,2]. Many patients experience benefits from immunotherapy and, for a portion of patients with advanced disease, these treatments can provide opportunities for long-term progression-free survival [2].

Sarcopenia is the loss of skeletal muscle mass (SMM) and strength, often caused by age or disease [3]. The measurement of SMM is often used to determine and diagnose sarcopenia in patients, and various techniques to measure SMM are available. The most commonly used measurements of SMM are on computed tomography (CT) or magnetic resonance imaging (MRI), especially in cancer patients where MRI and CT imaging are often part of standard care [3,4,5]. The prevalence of sarcopenia in cancer patients is high, with the majority of patients with advanced cancer showing low SMM [6,7]. Numerous studies have demonstrated the association between sarcopenia and increased risks of drug toxicity [8,9,10] or surgical complications [11,12,13], as well as decreased survival [10,13,14]. This predictive and prognostic relationship has been demonstrated in several patient populations, such as those diagnosed with melanoma [11], head and neck squamous cell carcinoma (HNSCC) [15], renal cell carcinoma (RCC) [8], gastrointestinal cancer [9,10,12], lung cancer [10,14], and hepatocellular carcinoma (HCC) [13].

The mechanism by which low SMM can cause increased risks of adverse events and decreased survival is still debated. One proposed explanation is that the interplay between sarcopenia and chronic systemic inflammation plays a critical role. This interplay may be of particular relevance in patients treated with immunotherapy, as these treatments utilise the patient’s own immune system, although the specific pathways by which this occurs still need to be investigated [16].

Most studies investigating the effect of sarcopenia in cancer patients have focused on single cancer types. In addition, there is a lack of consensus about which cutoff values to use in the diagnosis of low skeletal muscle mass. Cutoff values vary according to cancer types, ethnic groups, and populations, which complicates comparisons across studies. Therefore, there is a need for validated and standardised cutoff values to improve compatibility between studies and facilitate the extrapolation of results to broader populations [3,4].

This review and meta-analysis aim to investigate the prognostic effect of baseline sarcopenia in patients with solid tumours treated with immunotherapy. We will provide a comprehensive overview of all the current research on the prognostic effect of sarcopenia for overall survival (OS) and progression-free survival (PFS), focusing on immunotherapy treatments. By pooling the available data and performing a meta-analysis, we aim to quantify the prognostic effect of baseline sarcopenia and assess the consistency of this association across different solid cancer types.

## 2. Methods

### 2.1. Search Strategy

This systematic review was conducted in accordance with the Preferred Reporting Items for Systematic Reviews and Meta-Analyses (PRISMA) guidelines [17]. The PRISMA checklist is provided in Supplementary Materials Table S1. The review protocol was registered with PROSPERO (CRD420261328990). MEDLINE (via PubMed), EMBASE, Cochrane, and Scopus were searched from inception to 27 February 2025. Restrictions for English- or Dutch-language texts were used and, if available in the database, a restriction on the publication type was set to articles. The search terms included were immunotherapy, cancer, and sarcopenia, as well as synonyms and applicable MeSH terms.

### 2.2. Literature Screening

Studies were included if they met the following inclusion criteria: (1) the study must measure the influence of baseline low skeletal muscle mass on survival in patients with solid tumours treated with immunotherapy; and (2) muscle mass must be measured on CT or MRI and normalised for height as a skeletal muscle index (SMI). Reasons for exclusion were the following: (1) full texts were not in English or Dutch; (2) were animal or in vitro studies; (3) were reviews, conference abstracts, single case reports, or study protocols; or (4) were intervention studies, such as diet or exercise programmes.

Two researchers (L.F.J.H. & A.W.M.A.S.) independently assessed the studies based on their titles and abstract. Cases of disagreement were solved by consensus between the two researchers. Full-text screening then followed for potentially relevant studies.

### 2.3. Data Extraction

Data were extracted and collected from each of the included studies by two researchers (L.F.J.H. & L.A.D.). Cases of disagreement were solved by consensus between the two researchers. Data consisted of general study information (author and publication year), population details (i.e., population size, distribution men and women, cancer type), treatment details (i.e., immunotherapy type, first- or second-line and concomitant treatments), body composition evaluation (i.e., sarcopenia cutoff value, occurrence of low SMM, software used), and outcomes (hazard ratios). Only published data were included, as well as Supplementary Materials if provided on the publisher’s website.

### 2.4. Risk of Bias Assessment

The risk of bias for each included study was assessed using the Quality in Prognosis Studies (QUIPS) tool [18] by two researchers (L.F.J.H. & L.A.D.). Cases of disagreement were solved by consensus between the two researchers. The QUIPS tool uses six domains: study participation, study attrition, prognostic factor measurement, outcomes measurement, study confounding, and statistical analysis and reporting. Each domain is rated as low, moderate, or high risk of bias using the prompting items and considerations.

### 2.5. Data Analysis

Hazard ratios (HR) and 95% confidence intervals (95% CI) for OS and PFS were extracted from each of the included studies. If an adjusted HR was available, this was used for the meta-analysis, otherwise the unadjusted HR was included. Studies were excluded from the meta-analysis if (1) less than 90% of the patient population received immunotherapy; (2) the study used SMI as continuous parameter; or (3) the study used survival measures other than OS and/or PFS, such as two-year or three-year mortality.

The meta-analysis was performed using the meta and metafor packages in Rstudio (version 4.5.1). A restricted maximum likelihood (REML) random effects model was chosen because of the assumed heterogeneity between the included studies. The results were visualised in forest plots showing the HR and 95% CI. The Chi^2^ test and the *I*^2^ and τ^2^ statistic were used to assess heterogeneity.

Subgroup analysis was performed and stratified by the cancer type of the patient population. Studies were included in the subanalysis if 90% of the patient population were diagnosed with a specific cancer type and if a minimum of three studies were available. An additional subanalysis looked at treatment lines. Studies were included in this subanalysis if 90% of the patient population received immunotherapy as either first-line treatment or second-line treatment and beyond. *p*-values < 0.05 were considered statistically significant, unless otherwise specified.

## 3. Results

### 3.1. Search Results

**Figure 1:**
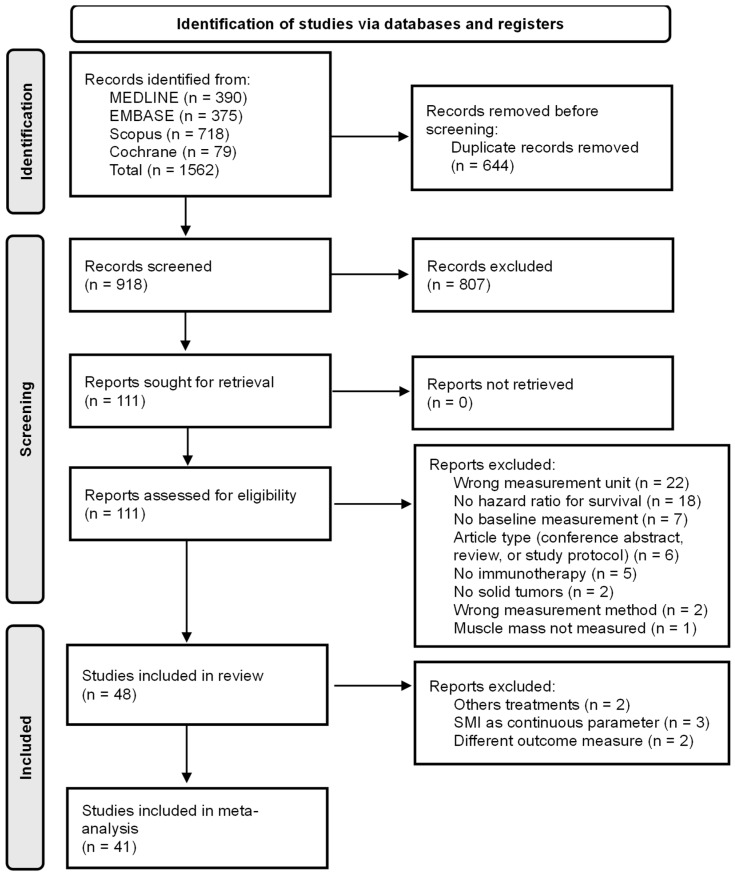
Preferred Reporting Items for Systematic Reviews and Meta-Analyses (PRISMA) flowchart detailing study selection.

The systematic literature search resulted in 1562 records. After removing 644 duplicates, the titles and abstracts of 918 studies were screened. After this initial screening by two researchers (L.F.J.H. and A.W.M.A.S.), 111 studies were retrieved for full-text screening. A total of 48 studies were included in the review [19,20,21,22,23,24,25,26,27,28,29,30,31,32,33,34,35,36,37,38,39,40,41,42,43,44,45,46,47,48,49,50,51,52,53,54,55,56,57,58,59,60,61,62,63,64,65,66], and 41 studies were included in the meta-analysis [19,20,21,22,23,25,26,28,29,30,32,33,34,35,36,37,39,41,42,43,45,46,47,48,49,50,51,52,53,54,55,56,57,58,59,60,61,62,63,65,66]. The screening, including the reasons for exclusion, is detailed in Figure 1.

### 3.2. Study Characteristics

Table 1 shows the study characteristics of the 48 included studies. There was a large range in population size from 28 to 800 patients. The most common cancer type was (non) small-cell lung carcinoma ((N)SCLC) and was included in 20 studies, followed by HCC included in 11 studies, RCC included in nine studies, melanoma included in six studies, HNSCC included in four studies, GC included in three studies, and urothelial carcinoma included in three studies. Median follow-up ranged from 6 to 32 months.

Not all studies specified which immunotherapy was used, but most defined the immunotherapy type, with the most common being programmed cell death protein 1 (PD-1) inhibitor, programmed cell death ligand 1 (PD-L1) inhibitor, and anti-cytotoxic T-lymphocyte-associated protein 4 (CTLA-4). Two studies [21,44] only stated that immune checkpoint inhibitors (ICIs) were used and did not further define the treatment. The studies which did specify the immunotherapy most commonly included pembrolizumab and nivolumab, followed by atezolizumab, ipilimumab, and camrelizumab.

Additional study characteristics can be found in the Supplementary Materials Table S2, which includes the mean or median age and BMI of the patient population, as well as details about the treatment line and concomitant treatments. Table S2 also provides more characteristics of the imaging used to determine sarcopenia. The vast majority of studies used a cross-sectional slide of CT on the level of L3 to measure skeletal muscle mass, except for Faron [31], who used L3-L4, and Wang [60], who used T5. The timing of the baseline CT was different per study, varying from within 6 months before the start of immunotherapy to the scan taken at the start of treatment.

### 3.3. Assessment of Risk of Bias Results

**Figure 2:**
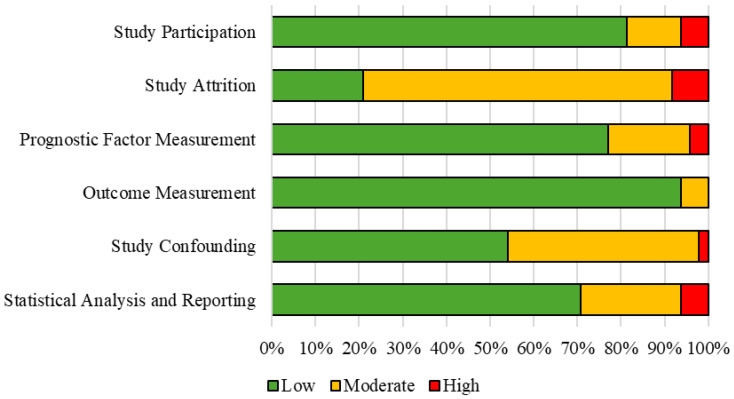
Quality in prognostic studies (QUIPS) assessment of the risk of bias, presented as a percentage of 48 included studies.

Table 2 and Figure 2 show the results of the QUIPS assessment of the risk of bias. The categories of study participation, prognostic factor measurement and outcome measurement were most often assessed with a low risk of bias in 39, 37 and 45 studies, respectively. The category of study attrition was assessed the most frequently with a moderate risk of bias in 34 studies, and a high risk of bias in four studies.

### 3.4. Prevalence of Sarcopenia

**Figure 3:**
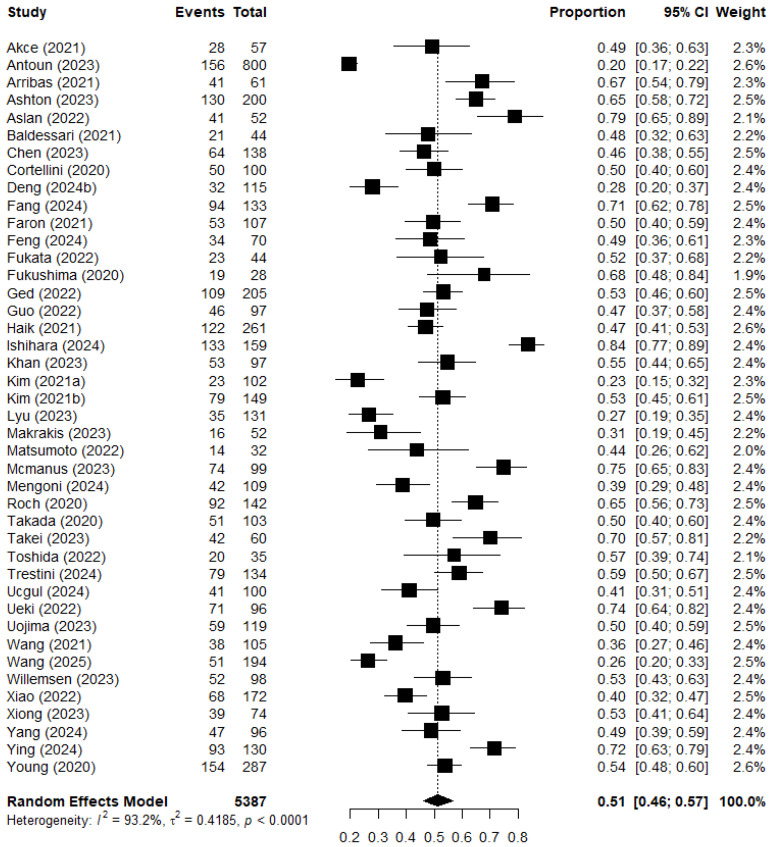
Forest plot of the pooled prevalence of sarcopenia as a proportion of the total population with a 95% confidence interval using a random effects model. The pooled prevalence is plotted with a black diamond.

The occurrence of sarcopenia, defined as low SMM, differed greatly between studies ranging from 19.5% to 83.6%. Figure 3 shows the pooled prevalence of sarcopenia as 51% (95% CI 46–57%) of the total population. The heterogeneity between the studies was high with an *I*^2^ of 93%.

Four studies used SMI as a continuous parameter and therefore did not dichotomise their population into sarcopenic and non-sarcopenic. The cutoff used to define the occurrence of sarcopenia also differed greatly. 32 studies referred to previously established cutoff values with the most common being those established by Martin et al. [14] at 43 cm^2^/m^2^ for men if BMI ≤ 24.9 kg/m^2^ or 53 cm^2^/m^2^ for men if BMI > 25 kg/m^2^ and 41 cm^2^/m^2^ for women, which were used in 13 of the 48 studies. Other commonly used cutoff values were those by Fearon et al. [67] in seven studies (55 cm^2^/m^2^ for men and 39 cm^2^/m^2^ for women); Prado et al. [10] in four studies (52.4 cm^2^/m^2^ for men and 38.5 cm^2^/m^2^ for women); and the Japan Society of Hepatology (JSP) [13] in three studies (42 cm^2^/m^2^ for men and 38 cm^2^/m^2^ for women). Studies which did not use previously established cutoff values employed differing methods to determine the cutoff value. These methods included setting the cutoff value at the median SMI [19,31,51,58] or at the 25th percentile [20,60] SMI and determining the optimal cutoff value through statistical analysis, such as with receiver operating characteristics (ROC) [26,28,29,36,53].

### 3.5. Association Between Low Skeletal Muscle Mass and Overall Survival

**Figure 4:**
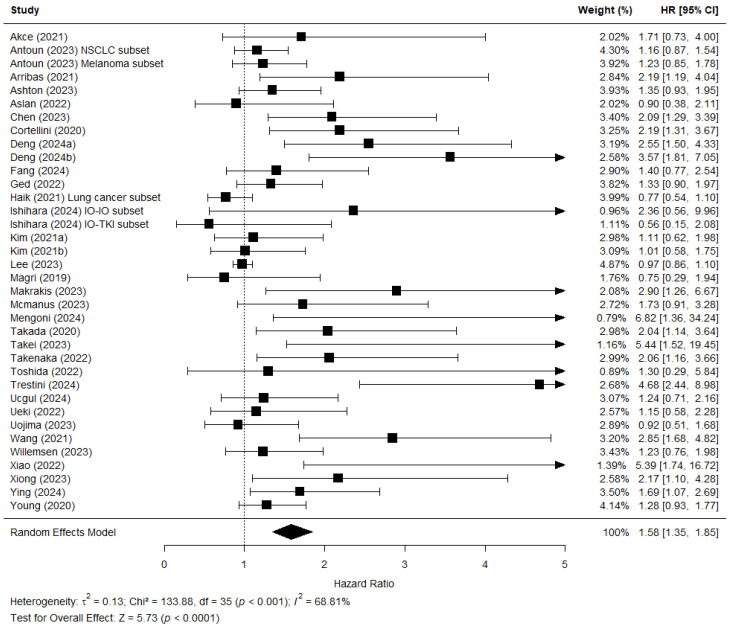
Forest plot showing the meta-analysis of the hazard ratios (HR) predicting the link between low skeletal muscle mass and overall survival (OS). The patient population of Antoun (2023) [20] was analysed using two subsets dividing patients with NSCLC and melanoma. Haik (2021) [37] only performed analysis of OS on their patients with lung cancer, creating the lung cancer subset. Ishihara (2024) [39] performed separate analyses for their subset treated with dual immunotherapy (IO-IO) and the subset treated with immunotherapy and TKI (IO-TKI). The combined effect of the studies is plotted with a black diamond.

Figure 4 shows the forest plot of the 34 studies that reported a hazard ratio for the effect of sarcopenia on OS. Antoun et al. [20] provided an analysis of two subsets, dividing their population of patients into those with NSCLC and those with melanoma. These subsets were included in the analysis separately. Haik et al. [37] only performed an analysis of OS for their subset of patients with lung carcinoma. Ishihara et al. [39] performed two separate analysis for their patient population treated with dual immunotherapy (IO-IO) and those treated with immunotherapy and TKI (IO-TKI). These two hazard ratios were entered into the analysis separately. The overall effect showed an association between baseline sarcopenia and OS (HR = 1.58; 95% CI 1.35–1.85; Z = 5.73 *p* < 0.0001). There was substantial heterogeneity between the studies in this analysis (Chi^2^ = 133.88; *p* < 0.001; *I*^2^ = 69%).

**Figure 5:**
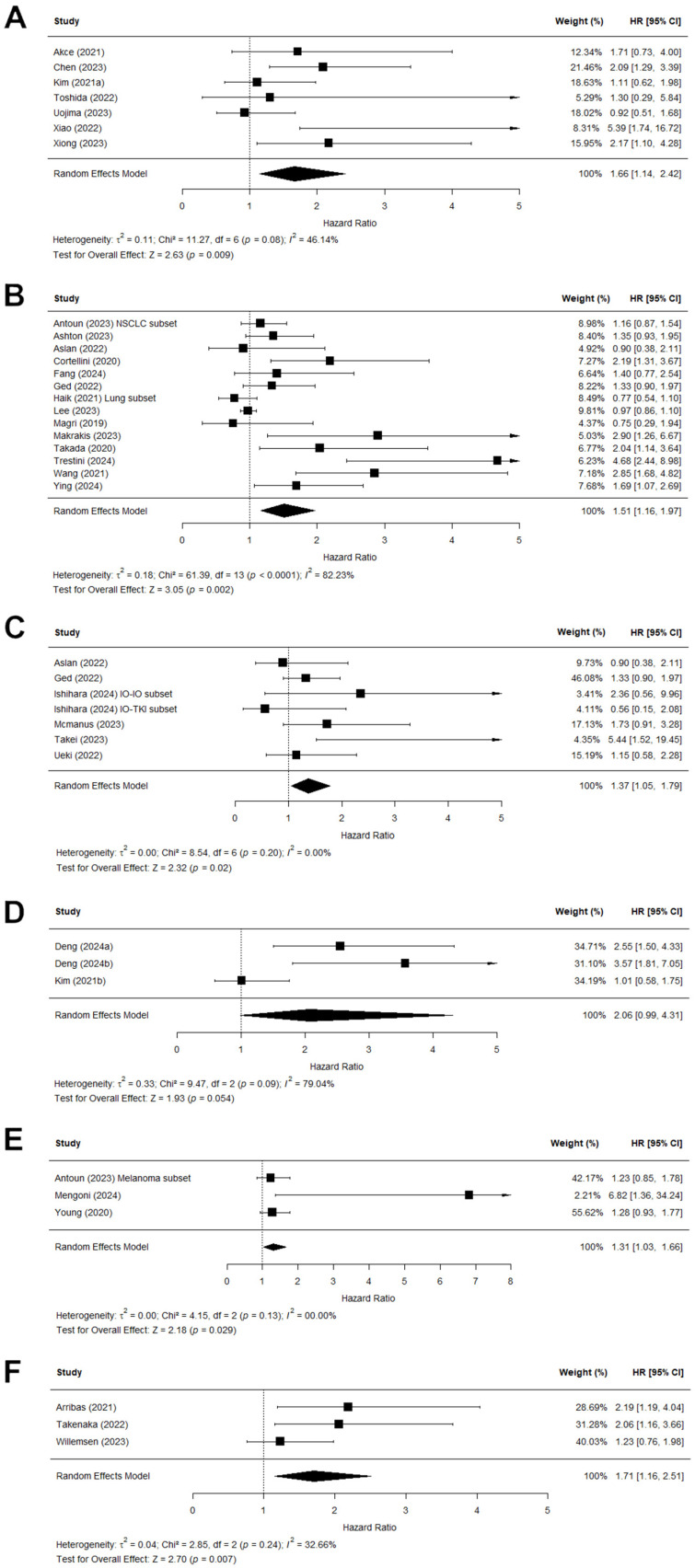
Forest plots showing the subanalyses of the hazard ratios (HR) predicting the link between low skeletal muscle mass and overall survival (OS), stratified by the cancer type. The combined effect of the studies is plotted with a black diamond. (**A**) The subanalyses of studies with hepatocellular carcinoma patients. (**B**) The subanalyses of studies with (non) small-cell lung carcinoma patients. (**C**) The subanalyses of studies with renal cell carcinoma patients. (**D**) The subanalyses of studies with gastric cancer patients. (**E**) The subanalyses of studies with melanoma patients. (**F**) The subanalyses of studies with head and neck squamous cell carcinoma patients.

To adjust for heterogeneity, further subset analysis was performed by dividing the population based on the cancer type. Figure 5 shows the forest plots for these subset analyses. All subanalyses showed an association between sarcopenia and OS with a pooled HR ranging between 1.31 and 2.06. All showed a significant test for overall effect, except for the gastric cancer (GC) subset which did show a trend towards significance (*p* = 0.054). The subanalyses of HCC, RCC, melanoma, and HNSCC showed a much lower level of heterogeneity, with the Chi^2^ no longer being significant and the *I*^2^ statistic ranging from 0% to 46%. The subanalysis of (N)SCLC still showed significant heterogeneity (Chi^2^ = 61.39; *p* < 0.0001; *I*^2^ = 82%) and the subanalysis of GC patients did not have a significant Chi^2^ but still high heterogeneity (Chi^2^ = 9.47; *p* = 0.09; *I*^2^ = 79%).

Additional subanalyses were performed with the studies stratified for treatment line. The studies with patients receiving immunotherapy as a first-line treatment showed a pooled HR of 2.67 with a significant test for overall effect (*p* = 0.005) (Figure S1A); heterogeneity remained present in this subanalysis (Chi^2^ = 12.57; *p* = 0.03; *I*^2^ = 61%). The studies with patients receiving immunotherapy as a second-line treatment or beyond showed a trend towards a significant overall effect (HR = 1.38; *p* = 0.051) (Figure S1B); heterogeneity was high in these studies (Chi^2^ = 39.48; *p* < 0.001; *I*^2^ = 80%).

### 3.6. Association Between Low Skeletal Muscle Mass and Progression-Free Survival

**Figure 6:**
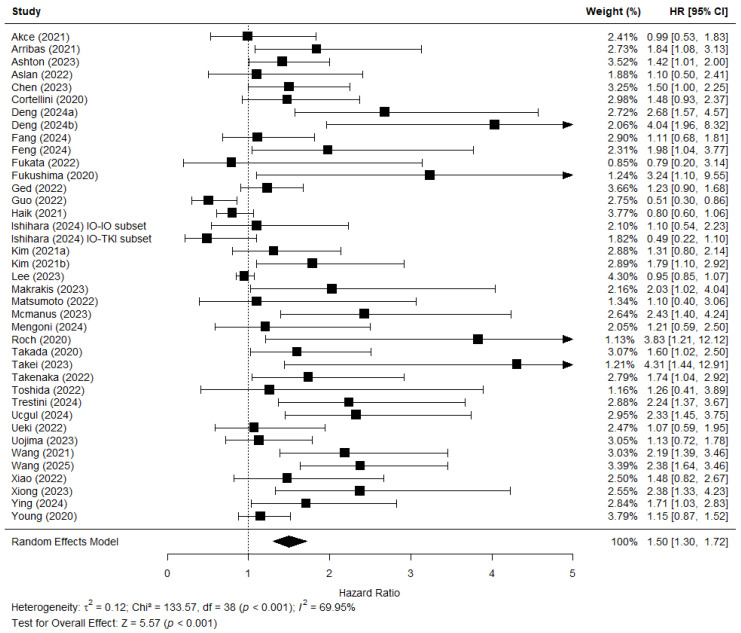
Forest plot showing the meta-analysis of the hazard ratios (HR) predicting the link between low skeletal muscle mass and progression-free survival (PFS). Haik (2021) [37] performed analysis of PFS on their entire population as well as the lung cancer subset. This plot shows the data for their entire population. The lung cancer subset is used in a later subanalysis. Ishihara (2024) [39] performed separate analyses for their subset treated with dual immunotherapy (IO-IO) and the subset treated with immunotherapy and TKI (IO-TKI). The combined effect of the studies is plotted with a black diamond.

Figure 6 shows the forest plot of 38 studies which reported a hazard ratio for the effect of sarcopenia on PFS. In the study by Ishihara et al. [39], two separate analyses were performed for patients treated with dual immunotherapy (IO-IO) and those treated with immunotherapy and TKI (IO-TKI). These two hazard ratios were entered into the analysis separately. The pooled effect demonstrated an association between sarcopenia and PFS (HR = 1.50; 95% CI 1.30–1.72; Z = 5.57 *p* < 0.001). There was considerable heterogeneity between the studies in this analysis (Chi^2^ = 133.57; *p* < 0.001; *I*^2^ = 70%).

**Figure 7:**
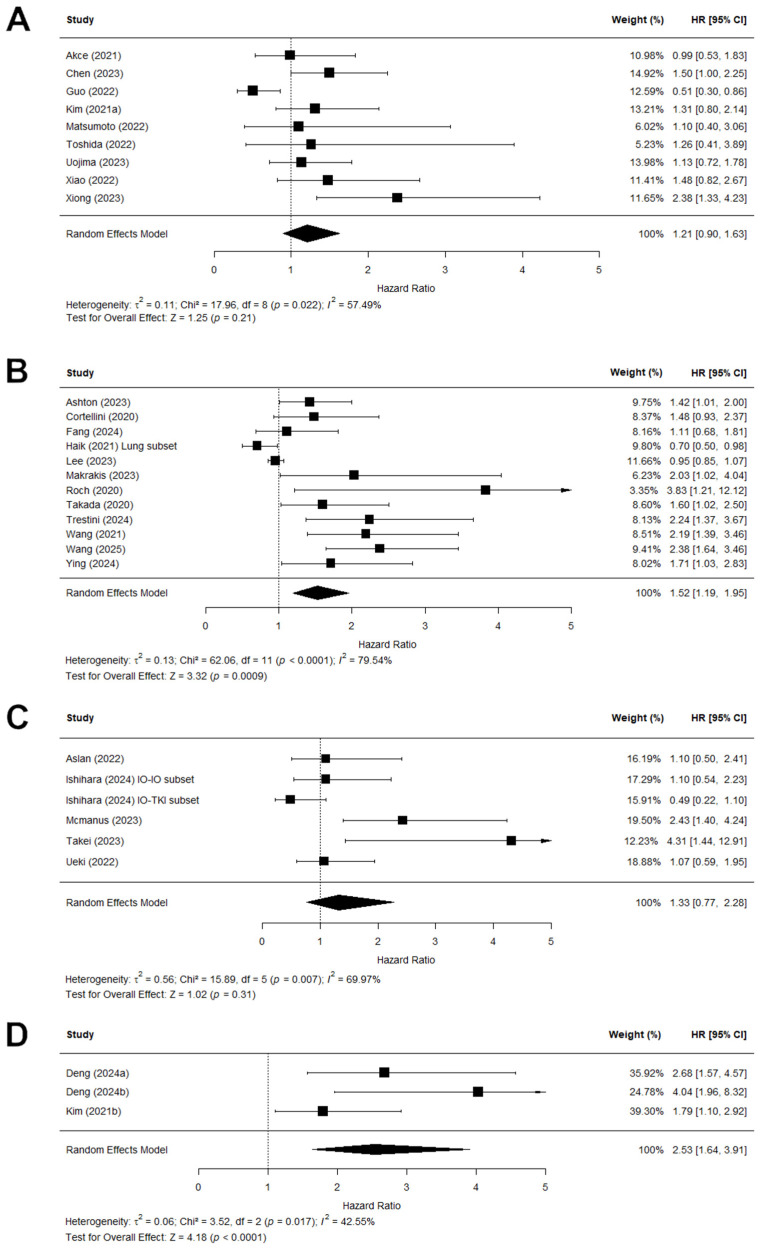
Forest plots showing the subanalyses of the hazard ratios (HR) predicting the link between low skeletal muscle mass and progression-free survival (PFS) stratified by cancer type. The combined effect of the studies is plotted with a black diamond. (**A**) The subanalyses of studies with hepatocellular carcinoma (HCC) patients. (**B**) The subanalyses of studies with (non) small-cell lung carcinoma ((N)SCLC) patients. Haik (2021) performed a separate analysis on a subset of their population consisting of patients with lung cancer. The results from that subanalysis are included here. (**C**) The subanalyses of studies with renal cell carcinoma (RCC) patients. Ishihara (2024) performed separate analyses for their subset treated with dual immunotherapy (IO-IO) and the subset treated with immunotherapy and TKI (IO-TKI). (**D**) The subanalyses of studies with gastric cancer (GC) patients.

Further subset analyses were performed by dividing the studies based on cancer type in order to adjust for the heterogeneity (Figure 7). These four subset analyses all showed a pooled HR ranging from 1.21 to 2.53, which demonstrated an association between sarcopenia and PFS; however, the test for overall effect was only significant in the subset of (N)SCLC and GC. Heterogeneity remained considerable in the subset analyses, with the Chi^2^ remaining significant in the HCC, (N)SCLC, and RCC subsets (*I*^2^ = 43–80%).

Additional subanalyses were peformed with the studies stratified for treatment line. Both subanalyses showed a pooled HR indicating the prognostic effect of sarcopenia for PFS and a significant overall effect. The studies with patients receiving immunotherapy as first-line treatment showed a pooled HR of 1.56 with a significant test for overall effect (*p* = 0.035) (Figure S1C). Heterogeneity remained present in this subanalysis (Chi^2^ = 17.93; *p* = 0.01; *I*^2^ = 63%). The studies with patients receiving immunotherapy as second-line treatment or beyond showed a pooled HR of 1.40 (*p* = 0.005) (Figure S1D). Heterogeneity remained present in this group (Chi^2^ = 40.92; *p* < 0.0001; *I*^2^ = 70%).

### 3.7. Adverse Events

Of the included studies, 17 studies [21,22,26,32,34,37,40,41,44,49,52,53,54,58,64,65,66] also looked at the association between low SMI and adverse events. The most common adverse events were skin toxicity, thyroid toxicity, pneumonia, arthritis, fatigue, and liver toxicity. The majority of the studies reported no significant association between low SMI and adverse events during immunotherapy treatment. The occurrence of adverse events had a wide range from 25% to 76% of patients experiencing adverse events. The majority of adverse events reported were grade 1 or grade 2 according to the Common Terminology Criteria for Adverse Events (CTCAE).

There were 14 studies that reported a similar occurrence of (severe) adverse events in patients with and without sarcopenia and found no significant difference between the two groups. Three studies reported a significant difference in the occurrence of adverse events between sarcopenic and non-sarcopenic patients. Feng et al. [32] showed a significant difference between the incidence of adverse events between the sarcopenia group and non-sarcopenia group (44% vs. 22%; *p* = 0.028); however, this difference was no longer significant when looked at severe adverse events (grade 4) (6% vs. 0%; *p* = 0.0513). Takei et al. [52] showed similar results with an adverse event incidence of 78% in patients with sarcopenia and 48% in patients without sarcopenia (*p* = 0.0463). This significant difference was not the case for severe adverse events (grade ≥ 3) (50% vs. 29% *p* = 0.144). Takenaka et al. [53] was the only study which found that severe adverse events (grade ≥ 3) occurred significantly more often in sarcopenic patients (OR: 6.00; 95% CI 1.04–34.61; *p* = 0.045).

## 4. Discussion

This review included 48 studies, of which 41 were also included in the meta-analysis. Among the 6822 patients in the included studies, sarcopenia prevalence was high, with a pooled prevalence of 51% of patients being sarcopenic. The meta-analysis showed a clear prognostic value of sarcopenia. The pooled HR of 1.58 demonstrated that sarcopenic patients with solid tumours who were treated with immunotherapy had a significantly lower overall survival. Additionally, the pooled HR of 1.50 showed lower progression-free survival in sarcopenic patients treated with immunotherapy. In the subanalyses per patient cancer types, this prognostic value remained clear. In the analysis for OS, when studies were grouped together by cancer types, this resulted in six subanalyses which all demonstrated lower OS for sarcopenic patients (HR 1.31–2.06). In the analysis for PFS, the stratification per cancer type resulted in four subanalyses which again supported the prognostic value of sarcopenia (HR 1.21–2.53). This supported previous research which had demonstrated the relationship between sarcopenia and prognosis [10,13,14]. In addition, it demonstrated that the prognostic value of sarcopenia was also present in patients treated with immunotherapy. Immunotherapy is a relatively newer treatment for many cancer types and the identification of predictive factors is therefore still in need of further investigation.

It is important to note that our analysis showed a consistent association between sarcopenia for both OS and PFS. Both analyses showed similar hazard ratios with Z-tests indicating a robust prognostic value of sarcopenia. In the further subanalysis, this consistency remained clear. The association between sarcopenia and OS was similar to the association between sarcopenia and PFS across cancer types. For example, in the subanalysis of (N)SCLC, the results of the OS analysis (HR 1.51, 95% CI 1.16–1.97; Z 3.05, *p* = 0.002) and PFS analysis (HR 1.52,95% CII 1.19–1.95; Z 3.32, *p* = 0.0009) were very similar. There was minimal variation across the cancer types in the effect size of the prognostic value of sarcopenia, both for OS (HR 1.31–2.06) and PFS (1.21–2.53). This variation suggested that the prognostic value of sarcopenia may be influenced by tumour-specific biological and clinical characteristics. Some factors that could influence these variations between cancer types are the differences in disease progression and staging, as well as possible differences in treatment regimens. In the subanalysis stratified for treatment line, we observed a higher prognostic value of sarcopenia for OS in patients treated with immunotherapy as a first-line treatment (HR 2.67, 95% CI 1.35–5.29; Z 2.82, *p* = 0.005), as opposed to second-line treatment or beyond (HR 1.56, 95% CI 1.03–2.36; Z 2.11, *p* = 0.035). This could possibly be explained by survivorship bias, as patients who are generally more frail would be less likely to successfully complete a first-line treatment and reach later stages of treatment, while those who are generally more fit would be more likely to receive second-line treatment or beyond.

This systematic review did not include adverse events as a primary outcome; however, some of the included articles did analyse the predictive value of sarcopenia for adverse events. Data analysis related to adverse events was not performed because there was insufficientdata and reporting methods varied between studies. The results presented were drawn from the 17 studies that also assessed adverse events. Most studies used CTCAE to categorise the adverse events, but some used other categories such as immune-related adverse events (irAE). Analyses were heterogeneous, with some focusing on severe events (grade > 3) and others examining any adverse event. Some reported the incidence of adverse events while others reported the numbers of patients who experienced adverse events. The majority of studies looked at differences in the occurrence of severe adverse events between the sarcopenic and non-sarcopenic groups. Overall, these analysis concluded that there were no differences in the occurrence of adverse events in sarcopenic vs. non-sarcopenic patients treated with immunotherapy.

There were some limitations in this review, as there was a high level of heterogeneity with an *I*^2^ of 69% (Figure 4) and 70% (Figure 5). This heterogeneity was reduced in subanalyses stratified by cancer type and treatment line. Because the heterogeneity remained moderate, other factors were also likely to have contributed to the level of heterogeneity. The heterogeneity could have been influenced by differences in populations, such as age, cancer stage, or treatment dosage. Another explanation for the high level of heterogeneity could be the different definitions of sarcopenia, which might also be responsible for the large variation in sarcopenia prevalence between the included studies. The measurement techniques varied slightly between studies, although all used CT imaging and most used the L3 level. Moreover, the cutoff values used to determine sarcopenia varied extensively. Many used the cutoff values as defined by Martin et al. [14] or other previously established values; however, several others also established their own cutoff values determined in various ways. The reason to choose different values was dependent on population details such as cancer type and country of origin; for example, several studies [25,41,42,44,47,54] chose to use cutoff values previously established in Asian populations, as the body composition of these patients can differ vastly from those in Western populations [68].

This review did not look at the relationship between sarcopenia and the objective response of patients treated with immunotherapy. The most common way to determine objective response was in accordance with the Response Evaluation Criteria in Solid Tumours (RECIST) guidelines [69]. There has been evidence that has shown that sarcopenia was predictive for lower changes in disease control and poor response in patients treated with immunotherapy [50,53]. Although RECIST can provide standardised criteria to determine the immunotherapy response, the means to quantify this differed in studies, with some reporting response rates, percentages of patients who achieved complete, partial response, or stable disease, and other disease control rates. This difference in reporting complicated comparing these in a meta-analysis; therefore, this review used PFS as an appropriate alternative measure to establish disease control and treatment response in accordance with RECIST guidelines [69].

The mechanisms behind the connection between sarcopenia and worsened survival has been debated. Sarcopenia, malnutrition, and cachexia have been linked to chronic inflammation [16]. Chronic inflammation in turn can contribute to muscle breakdown through cytokines such as interleukin-6 (IL-6) and tumour necrosis factor alpha (TNF-α) [70]. In addition, skeletal muscle cells can produce anti-inflammatory cytokines, such as IL-15, with an increased production during exercise. These anti-inflammatory cytokines decrease the activity of TNF-α and thereby interrupt further muscle breakdown [70]. Skeletal muscle cell regeneration can also be stimulated by chemotactic factors in the intercellular content released by muscle cells during exercise [16]. In summary, sarcopenia has been linked to chronic inflammation which can cause muscle breakdown, and the decreased muscle mass can then cause lower levels of anti-inflammatory factors. This is especially of interest in patients treated with immunotherapy, as chronic inflammation has also been linked to immunotherapy resistance because the increased levels of cytokines can cause T-cell exhaustion and T-cell exclusion from the tumour site [16,70]. There is a need to further understand the exact mechanisms which can contribute to the connection between sarcopenia and lower survival, as well as immunotherapy resistance. A further understanding will help in the development of correct intervention approaches to treat sarcopenia and improve prognosis of sarcopenic patients.

Recently, there has been research into appropriate interventions such as supplements, dietary advice, and exercise to prevent or treat sarcopenia in cancer patients [71]; however, this research is ongoing and there is no consensus yet on how to properly treat sarcopenia. There has also been recent research about the adaptation of treatment based on the presence of sarcopenia—this could be a change in treatment type, schedule, or dosing [72]. One of the difficulties faced in this new research is the lack of universal cutoff values for the diagnosis of sarcopenia. Additionally, the treatment changes are most likely different based on cancer type and treatment agent. The results from this review and meta-analysis further support the need for research on this topic, preferably with randomised controlled trials for specific intervention regimens, such as nutrition and exercise, to improve the prognosis of sarcopenic patients.

## 5. Conclusions

This review and meta-analysis demonstrated that baseline sarcopenia, defined as the presence of low skeletal muscle mass, is a significant adverse prognostic factor for both overall survival (HR 1.58; 95% CI 1.35–1.85; *p* < 0.0001) and progression-free survival (HR 1.50; 95% CI 1.30–1.72; *p* < 0.001) across cancer types with solid tumours treated with immunotherapy. In addition, the underlying mechanism of sarcopenia and its connection with the immune response and survival requires additional research. Prospective intervention studies are required to establish the care, such as nutrition and exercise programmes, needed to improve the prognosis of sarcopenic patients.

## Figures and Tables

**Table 1:** Study characteristics.

Author (Year)	Country	n	Men n (%) Women n (%)	Cancer Type	Immunotherapy	Cutoff Sarcopenia/Low SMM in cm^2^/m^2^	Occurrence Sarcopenia n (%) ^a^	Median Follow-Up	Median OS	Median PFS
Akce (2021) [19]	USA	57	44 (77%) 13 (23%)	Advanced HCC	PD-1 inhibitor	<43 for men and <39 for women	28 (49.1%)	6 mos.	-	-
Antoun (2023) [20]	France	800	457 (57%) 343 (43%)	411 (51%) melanoma 389 (49%) NSCLC	408 (51%) pembrolizumab 361 (45%) nivolumab 15 (2%) nivolumab + ipilimumab	Sex-specific cutoff at 25th percentile	156 (19.5%)	25.4 mos.	18.9 mos.	9.1 mos.
Arribas (2021) [21]	Spain	61	52 (85%) 9 (15%)	Recurrent or metastatic HNSCC	ICI	According to Martin ^1^	41 (67.2%)	9 mos.	-	-
Ashton (2023) [22]	France	200	126 (63%) 74 (37%)	Metastatic NSCLC	147 (74%) nivolumab 53 (26%) pembrolizumab	According to Prado ^2^	130 (67%)	-	-	-
Aslan (2022) [23]	Turkey	52	38 (73%) 14 (27%)	Metastatic RCC	Nivolumab	EWGSOP cutoff	41 (79%)	11.4 mos.	-	-
Baldessari (2021) [24]	Italy	44	26 (59%) 18(41%)	Advanced NSCLC	Pembrolizumab	According to Fearon ^3^	21 (52.5%)	265 days	9.01 mos.	5.6 mos.
Chen (2023) [25]	Taiwan	138	120 (87%) 18 (13%)	Advanced HCC	105 (76.1%) PD-1 2 (1.4%) anti-CTLA-4 26 (18.8%) PD-1 + anti-CTLA-4 5 (3.6%) others	<40.8 for men and <34.9 for women	64 (46.4%)	30.5 mos.	16.5 mos.	4.8 mos.
Cortellini (2020) [26]	Italy	100	67 (67%) 33 (33%)	Advanced cancer: 46 (46%) NSCLC 27 (27%) melanoma 15 (15%) RCC 12 (12%) others	91 (91%) PD-1 inhibitor 9 (9%) PD-L1 inhibitor	For men ≤50.2 if BMI ≥ 25 kg/m^2^ or ≤48.4 if BMI < 25 kg/m^2^; For women ≤59.6 if BMI ≥ 25 kg/m^2^ or ≤36.9 if BMI < 25 kg/m^2^	50 (50%)	20.3 mos.	10.4 mos.	3.7 mos.
Crombe (2020) [27]	France	117	62 (53%) 55 (47%)	Metastatic solid tumours: 65 (56%) NSCLC 14 (12%) STS 8 (7%) PRAD 6 (5%) UC 24 (20%) others	89 (76%) PD-1 inhibitor 12 (10%) PD-L1 inhibitor 16 (14%) PD-L1 inhibitor + anti-CTLA4	Continuous parameter	Median 44.84 cm^2^/m^2^	-	-	125 days
Deng (2024a) [28]	China	124	96 (77%) 28 (23%)	GC	PD-1 or PD-L1 inhibitor	<27.36 for men and <31.10 for women	-	-	-	-
Deng (2024b) [29]	China	115	89 (77%) 26 (23%)	GC	PD-1 or PD-L1 inhibitor	<27.36 for men and <31.10 for women	32 (27.4%)	-	-	-
Fang (2024) [30]	China	133	114 (86%) 19 (14%)	SCLC	72 (54%) PD-1 inhibitor 61 (46%) PD-L1 inhibitor	According to Fearon ^3^	94 (70.7%)	552 days	331 days	169 days
Faron (2021) [31]	Germany	107	70 (65%) 37 (35%)	Metastatic melanoma	70 (65%) nivolumab or pembrolizumab 20 (19%) nivolumab + ipilimumab 17 (16%) ipilimumab	<51.9 for men and <41.0 for women	53(49.5%)	-	3 year mortality 28%	-
Feng (2024) [32]	China	70	60 (86%) 10 (14%)	NSCLC	PD-1 or PD-L1 inhibitor	According to Martin ^1^	34 (48.6%)	-	-	10.2 mos.
Fukata (2022) [33]	Japan	44	30 (68%) 14 (32%)	Advanced UC	Pembrolizumab	According to Fearon ^3^	23 (53%)	13.2 mos.	-	-
Fukushima (2020) [34]	Japan	28	19 (68%) 9 (32%)	Advanced UC	Pembrolizumab	According to Martin ^1^	19 (68%)	6 mos.	12 mos.	4 mos.
Ged (2022) [35]	USA	205	152 (74%) 53 (26%)	Metastatic clear cell RCC	134 (65%) PD-1 or PD-L1 inhibitor 64 (31%) PD-1 inhibitor + anti-CTLA-4 7 (4%) PD-1 + PD-L1 inhibitor	According to Fearon ^3^	109 (53%)	31.2 mos.	-	-
Guo (2022) [36]	China	97	79 (81%) 18 (19%)	Advanced HCC	Camrelizumab	<37.7 for men and <34.3 for women	46 (47.4%)	-	-	5.30 mos.
Haik (2021) [37]	France	261	198 (76%) 63 (24%)	Metastatic solid tumours: 179 (69%) lung cancer 50 (19%) RCC 24 (9%) HNSCC 8 (3%) bladder cancer	192 (74%) PD-1 inhibitor 43 (16%) PD-L1 inhibitor 3 (1%) anti-CTLA-4 23 (9%) ICI combinations	According to Martin ^1^	122 (47%)	-	-	28.4 weeks
Imai (2025) [38]	Japan	214	171 (80%) 43 (20%)	HCC	79 (37%) sorafenib 76 (36%) lenvatinib 43 (20%) atezolizumab + bevacizumab 6 (3%) cabozantinib 5 (2%) temelimumab + durvalumab 3 (1%) ramcirumab 1 (<1%) regorafenib	Continuous parameter	Median 42.96 cm^2^/m^2^	-	-	-
Ishihara (2024) [39]	Japan	159	115 (72%) 44 (28%)	Advanced RCC	84 (53%) nivolumab + ipilimumab 75 (47%) pembrolizumab or nivolumab or avelumab	According to Fearon ^3^	133 (83.6%)	17.8 mos.	-	-
Khan (2023) [40]	Australia	97	55 (57%) 42 (43%)	Advanced lung cancer	57 (58.8%) nivolumab 24 (24.7%) pembrolizumab 16 (16.5%) atezolizumab	According to Martin ^1^	53 (54.6%)	-	15.4 mos.	11.3 mos.
Kim (2021a) [41]	South Korea	102	87 (85%) 15 (15%)	HCC	Nivolumab	According to JSP ^4^	23 (22.5%)	21.9 mos.	5.1 mos.	1.7 mos.
Kim (2021b) [42]	South Korea	149	93 (62%) 56 (38%)	Microsatellite-stable GC	83 (56%) pembrolizumab 66 (44%) nivolumab	≤49 for men and ≤31 for women	79 (53.0%)	20.3 mos.	4.9 mos.	1.9 mos.
Lee (2023) [43]	South Korea	820	630 (77%) 190 (23%)	Advanced NSCLC	417 (51%) pembrolizumab 271 (33%) atezolizumab 132 (16%) nivolumab	Continuous parameter	-	6.9 mos.	6.9 mos.	2.7 mos.
Lyu (2023) [44]	China	131	77 (59%) 54 (41%)	Advanced NSCLC	54 (41%) ICIs 77 (59%) EGFR-TKI	<40.2 for men and <31.6 for women	35 (26.7%)	-	-	-
Magri (2019) [45]	Israel	46	28 (61%) 18 (39%)	Advanced NSCLC	Nivolumab	Continuous parameter	Mean 42.43 cm^2^/m^2^	22 mos.	6.89	-
Makrakis (2023) [46]	Greece	52	43 83%) 9 (17%)	Non-oncogene driven metastatic NSCLC	34 (65%) nivolumab 16 (31%) pembrolizumab 2 (4%) atezolizumab	According to Fearon ^3^	16 (30.8%)	9.9 mos.	-	-
Matsumoto (2022) [47]	Japan	32	19 (59%) 13 (41%)	HCC	Atezolizumab + Bevacizumab	According to JSP ^4^	14 (44%)	10.4 mos.	-	6.5 mos.
Mcmanus (2023) [48]	USA	99	73 (74%) 26 (26%)	Metastatic RCC	Ipilimumab + nivolumab	According to Fearon ^3^	74 (74.7%)	25.7 mos.	39.8 mos.	-
Mengoni (2024) [49]	Germany	109	61 (56%) 48 (44%)	Stage IIB–IV melanoma	63 (58%) pembrolizumab 41 (38%) nivolumab 5 (5%) other PD-1	According to Prado ^2^	42 (38.5%)	32.7 mos.	-	-
Roch (2020) [50]	France	142	93 (65%) 49 (35%)	NSCLC	Pembrolizumab or nivolumab	According to Prado ^2^	92 (65.71%)	-	-	-
Takada (2020) [51]	Japan	103	84 (82%) 19 (18%)	NSCLC	71 (69%) nivolumab 32 (31%) pembrolizumab	<25.63 for men and <21.73 for women	51 (50%)	228 days	-	-
Takei (2023) [52]	Japan	60	46 (77%) 14 (23%)	Metastatic RCC	34 (57%) nivolumab + ipilimumab 12 (20%) avelumab 10 (17%) pembrolizumab 4 (7%) nivolumab	According to Martin ^1^	42 (70%)	15 mos.	-	-
Takenaka (2022) [53]	Japan	114	85 (75%) 29 (25%)	HNSCC	Nivolumab	<39.84 for men and <35.92 for women	Median 41.27 cm^2^/m^2^	23.1 mos.	-	-
Toshida (2022) [54]	Japan	35 ^b^	28 (80%) 7 (20%)	HCC	Atezolizumab + bevacizumab	According to JSP ^4^	20 (57.1%)	-	-	-
Trestini (2024) [55]	Italy	134	93 (69%) 41 (31%)	NSCLC	Pembrolizumab	According to Martin ^1^	79 (59.0%)	12 mos.	19.3 mos.	7.4 mos.
Ucgul (2024) [56]	Turkey	100	69 (69%) 31 (31%)	25 (25%) melanoma 22 (22%) RCC 18 (18%) NSCLC 35 (35%) other	70 (70%) nivolumab 14 (14%) Atezolizumab 6 (6%) pembrolizumab 10 (10%) Ipilimumab	<45.4 for men and <34.4 for women	41 (41%)	-	-	-
Ueki (2022) [57]	Japan	96	71 (74%) 25 (26%)	Metastatic RCC	Nivolumab	According to Martin ^1^	71 (74%)	9.7 mos.	26.4 mos.	
Uojima (2023) [58]	Japan	119	98 (78%) 21 (22%)	HCC	Atezolizumab + bevacizumab	<42.2	59 (50%)	-	Mean 512 days	Mean 209 days
Wang (2021) [59]	China	105	73 (70%) 32 (30%)	Stage IV NSCLC	35 (33%) nivolumab 46 (44%) pembrolizumab 24 (23%) camrelizumab	According to Martin ^1^	38 (36.2%)	14.73 mos.	14.7 mos.	-
Wang (2025) [60]	China	194 ^c^	150 (77%) 44 (23%)	NSCLC	PD-1 or PD-L1 inhibitor	Sex-specific cutoff at 25th percentile	51 (26.3%)	18.7 mos.	-	12 mos.
Willemsen (2023) [61]	Netherlands Belgium	98	83 (85%) 15 (15%)	Recurrent and/or metastatic HNSCC	PD-1 or PD-L1 inhibitor	According to Martin ^1^	52 (53%)	9 mos.	-	-
Xiao (2022) [62]	China	172	149 (87%) 23 (13%)	Primary liver cancer	PD-1 or PD-L1 inhibitor	According to Martin ^1^	68 (39.5%)	9 mos.	-	-
Xiong (2023) [63]	China	74	63 (85%) 11 (15%)	HCC	72 (97%) PD-1 inhibitor 2 (3%) PD-L1 inhibitor	According to Martin ^1^	39 (52.7%)	16.1 mos.	18.4 mos.	6.7 mos.
Yang (2024) [64]	China	96	87 (91%) 9 (9%)	HCC	Toripalimab or camrelizumab	<45.37 for men and <36.33 for women	47 (49.0%)	680 days	553 days	-
Ying (2024) [65]	China	130	130 (100%) men	Squamous cell lung cancer	PD-1 inhibitor	According to Prado ^2^	93 (71.5%)	Mean 20.8 mos.	13.3 mos.	-
Young (2020) [66]	USA	287	184 (64%) 103 (36%)	Metastatic melanoma	186 (65%) pembrolizumab 62 (22%) ipilimumab + nivolumab 32 (11%) nivolumab 7 (2%) atezolizumb	According to Martin ^1^	154 (53.7%)	519 days	-	-

^a^ If the study did not report the number of patients with sarcopenia/low SMI, the median or mean SMI was reported where available. ^b^ Toshida (2022) [54] included 98 patients, of which 35 were treated with immunotherapy. All other patients were treated with lenvatinib. The results were reported separately; therefore, this review only included the subset treated with immunotherapy. ^c^ Wang (2025) [60] included 242 patients, of which 48 were in a validation cohort. The general analysis was performed on the discovery cohort of 194 patients; therefore, this review only included the discovery cohort. ^1^ According to Martin et al., the cutoff was 43 cm^2^/m^2^ if BMI ≤ 24.9 kg/m^2^ or 53 cm^2^/m^2^ if BMI > 25 kg/m^2^ for men; and 41 cm^2^/m^2^ for women [14]. ^2^ According to Prado et al. the cutoff was 52.4 cm^2^/m^2^ for men and 38.5 cm^2^/m^2^ for women [10]. ^3^ According to Fearon et al., the cutoff was 55 cm^2^/m^2^ for men and 39 cm^2^/m^2^ for women [67]. ^4^ According to Japan Society of Hepatology (JSP) guidelines used the cutoff of 42 cm^2^/m^2^ for men and 38 cm^2^/m^2^ for women [13]. BMI = body mass index; CTLA-4 = cytotoxic T-lymphocyte-associated protein 4; EGFR = epidermal growth factor receptor; EWGSOP = European Working Group on Sarcopenia in Older People; GC = gastric cancer; HCC = hepatocellular carcinoma; HNSCC = head and neck squamous cell carcinoma; ICI = immune checkpoint inhibitor; mos. = months; NSCLC = non-small-cell lung carcinoma; OS = overall survival; PD-1 = programmed cell death protein 1; PD-L1 = programmed cell death ligand 1; PFS = progression-free survival; PRAD = prostate adenocarcinoma; RCC = renal cell carcinoma; SCLC = small-cell lung carcinoma; SMM = skeletal muscle mass; STS = soft tissue sarcoma; TKI = tyrosine kinase inhibitor; UC = urothelial carcinoma.

**Table 2:** Quality in prognostic studies (QUIPS) assessment of risk of bias.

	Study Participation	Study Attrition	Prognostic Factor Measurement	Outcome Measurement	Study Confounding	Statistical Analysis and Reporting
Akce (2021)	⬤	⬤	⬤	⬤	⬤	⬤
Antoun (2023)	⬤	⬤	⬤	⬤	⬤	⬤
Arribas (2021)	⬤	⬤	⬤	⬤	⬤	⬤
Ashton (2023)	⬤	⬤	⬤	⬤	⬤	⬤
Aslan (2022)	⬤	⬤	⬤	⬤	⬤	⬤
Baldessari (2021)	⬤	⬤	⬤	⬤	⬤	⬤
Chen (2023)	⬤	⬤	⬤	⬤	⬤	⬤
Cortellini (2020)	⬤	⬤	⬤	⬤	⬤	⬤
Crombe (2020)	⬤	⬤	⬤	⬤	⬤	⬤
Deng (2024a)	⬤	⬤	⬤	⬤	⬤	⬤
Deng (2024b)	⬤	⬤	⬤	⬤	⬤	⬤
Fang (2024)	⬤	⬤	⬤	⬤	⬤	⬤
Faron (2021)	⬤	⬤	⬤	⬤	⬤	⬤
Feng (2024)	⬤	⬤	⬤	⬤	⬤	⬤
Fukata (2022)	⬤	⬤	⬤	⬤	⬤	⬤
Fukushima (2020)	⬤	⬤	⬤	⬤	⬤	⬤
Ged (2022)	⬤	⬤	⬤	⬤	⬤	⬤
Guo (2022)	⬤	⬤	⬤	⬤	⬤	⬤
Haik (2021)	⬤	⬤	⬤	⬤	⬤	⬤
Imai (2025)	⬤	⬤	⬤	⬤	⬤	⬤
Ishihara (2024)	⬤	⬤	⬤	⬤	⬤	⬤
Khan (2023)	⬤	⬤	⬤	⬤	⬤	⬤
Kim (2021a)	⬤	⬤	⬤	⬤	⬤	⬤
Kim (2021b)	⬤	⬤	⬤	⬤	⬤	⬤
Lee (2023)	⬤	⬤	⬤	⬤	⬤	⬤
Lyu (2023)	⬤	⬤	⬤	⬤	⬤	⬤
Magri (2019)	⬤	⬤	⬤	⬤	⬤	⬤
Makrakis (2023)	⬤	⬤	⬤	⬤	⬤	⬤
Matsumoto (2022)	⬤	⬤	⬤	⬤	⬤	⬤
Mcmanus (2023)	⬤	⬤	⬤	⬤	⬤	⬤
Mengoni (2024)	⬤	⬤	⬤	⬤	⬤	⬤
Roch (2020)	⬤	⬤	⬤	⬤	⬤	⬤
Takada (2020)	⬤	⬤	⬤	⬤	⬤	⬤
Takei (2023)	⬤	⬤	⬤	⬤	⬤	⬤
Takenaka (2022)	⬤	⬤	⬤	⬤	⬤	⬤
Toshida (2022)	⬤	⬤	⬤	⬤	⬤	⬤
Trestini (2024)	⬤	⬤	⬤	⬤	⬤	⬤
Ucgul (2024)	⬤	⬤	⬤	⬤	⬤	⬤
Ueki (2022)	⬤	⬤	⬤	⬤	⬤	⬤
Uojima (2023)	⬤	⬤	⬤	⬤	⬤	⬤
Wang (2021)	⬤	⬤	⬤	⬤	⬤	⬤
Wang (2025)	⬤	⬤	⬤	⬤	⬤	⬤
Willemsen (2023)	⬤	⬤	⬤	⬤	⬤	⬤
Xiao (2022)	⬤	⬤	⬤	⬤	⬤	⬤
Xiong (2023)	⬤	⬤	⬤	⬤	⬤	⬤
Yang (2024)	⬤	⬤	⬤	⬤	⬤	⬤
Ying (2024)	⬤	⬤	⬤	⬤	⬤	⬤
Young (2020)	⬤	⬤	⬤	⬤	⬤	⬤

⬤ Low risk of bias; ⬤ moderate risk of bias; ⬤ high risk of bias.

## Data Availability

No new data were created or analysed in this study.

## Supplementary Materials

The following supporting information can be downloaded at: https://www.mdpi.com/article/10.3390/jcm15072720/s1, Table S1: PRISMA checklist; Table S2: Supplementary study characteristics; Figure S1: Forest plots showing the subanalyses of the hazard ratios (HR) predicting the link between low skeletal muscle mass and survival stratified by first-line treatment and second-line treatment or beyond.

## Abbreviations

The following abbreviations are used in this manuscript:

HR	Hazard Ratio
OS	Overall Survival
PFS	Progression-Free Survival
CT	Computed Tomography
MRI	Magnetic Resonance Imaging
SMM	Skeletal Muscle Mass
HNSCC	Head and Neck Squamous Cell Carcinoma
RCC	Renal Cell Carcinoma
HCC	Hepatocellular Carcinoma
PRISMA	Preferred Reporting Items for Systematic Reviews and Meta-Analyses
SMI	Skeletal Muscle Index
QUIPS	Quality in Prognosis Studies
(N)SCLC	(Non) Small-Cell Lung Carcinoma
PD-1	Programmed Cell Death Protein 1
PD-L1	Programmed Cell Death Ligand 1
CTLA-4	Cytotoxic T-Lymphocyte-Associated Protein 4
ICI	Immune Checkpoint Inhibitor
JSP	Japan Society of Hepatology
ROC	Receiver Operating Characteristics
GC	Gastric Cancer
CTCAE	Common Terminology Criteria for Adverse Events
irAE	Immune-Related Adverse Events
RECIST	Response Evaluation Criteria in Solid Tumours
IL-6	Interleukin 6
TNF- α	Tumour Necrosis Factor Alpha
