# Supplementary material for: The Prognostic Effect of Sarcopenia in Solid Cancers Treated with Immunotherapy: A Systematic Review and Meta-Analysis
Source: J Clin Med. 2026 Apr 3;15(7):2720. doi: 10.3390/jcm15072720 (PMC13073668; doi:10.3390/jcm15072720)
Supplement: Supplementary file 1 [file jcm-15-02720-s001.zip › jcm-4183065-supplementary.pdf]

Supplementary Materials

Supplementary table S1. PRISMA checklist

Section and Topic	Item #	Checklist item	Location where item is reported
TITLE			
Title	1	Identify the report as a systematic review.	Page 1
ABSTRACT			
Abstract	2	See the PRISMA 2020 for Abstracts checklist.	Page 1
INTRODUCTION			
Rationale	3	Describe the rationale for the review in the context of existing knowledge.	Page 1-2
Objectives	4	Provide an explicit statement of the objective(s) or question(s) the review addresses.	Page 1-2
METHODS			
Eligibility criteria	5	Specify the inclusion and exclusion criteria for the review and how studies were grouped for the syntheses.	Page 3
Information sources	6	Specify all databases, registers, websites, organisations, reference lists and other sources searched or consulted to identify studies. Specify the date when each source was last searched or consulted.	Page 3
Search strategy	7	Present the full search strategies for all databases, registers and websites, including any filters and limits used.	Page 3
Selection process	8	Specify the methods used to decide whether a study met the inclusion criteria of the review, including how many reviewers screened each record and each report retrieved, whether they worked independently, and if applicable, details of automation tools used in the process.	Page 3
Data collection process	9	Specify the methods used to collect data from reports, including how many reviewers collected data from each report, whether they worked independently, any processes for obtaining or confirming data from study investigators, and if applicable, details of automation tools used in the process.	Page 3
Data items	10a	List and define all outcomes for which data were sought. Specify whether all results that were compatible with each outcome domain in each study were sought (e.g. for all measures, time points, analyses), and if not, the methods used to decide which results to collect.	Page 3-4
	10b	List and define all other variables for which data were sought (e.g. participant and intervention characteristics, funding sources). Describe any assumptions made about any missing or unclear information.	Page 3-4; 6-11

Section and Topic	Item #	Checklist item	Location where item is reported
Study risk of bias assessment	11	Specify the methods used to assess risk of bias in the included studies, including details of the tool(s) used, how many reviewers assessed each study and whether they worked independently, and if applicable, details of automation tools used in the process.	Page 3
Effect measures	12	Specify for each outcome the effect measure(s) (e.g. risk ratio, mean difference) used in the synthesis or presentation of results.	Page 6-7; Supplementary information
Synthesis methods	13a	Describe the processes used to decide which studies were eligible for each synthesis (e.g. tabulating the study intervention characteristics and comparing against the planned groups for each synthesis (item #5)).	Page 3-4
	13b	Describe any methods required to prepare the data for presentation or synthesis, such as handling of missing summary statistics, or data conversions.	Page 3-4
	13c	Describe any methods used to tabulate or visually display results of individual studies and syntheses.	Page 3-4
	13d	Describe any methods used to synthesize results and provide a rationale for the choice(s). If meta-analysis was performed, describe the model(s), method(s) to identify the presence and extent of statistical heterogeneity, and software package(s) used.	Page 3-4
	13e	Describe any methods used to explore possible causes of heterogeneity among study results (e.g. subgroup analysis, meta-regression).	Page 3-4
	13f	Describe any sensitivity analyses conducted to assess robustness of the synthesized results.	Page 3-4
Reporting bias assessment	14	Describe any methods used to assess risk of bias due to missing results in a synthesis (arising from reporting biases).	Page 3-4
Certainty assessment	15	Describe any methods used to assess certainty (or confidence) in the body of evidence for an outcome.	Page 3-4
RESULTS			
Study selection	16a	Describe the results of the search and selection process, from the number of records identified in the search to the number of studies included in the review, ideally using a flow diagram.	Page 4
	16b	Cite studies that might appear to meet the inclusion criteria, but which were excluded, and explain why they were excluded.	Page 4-5
Study characteristics	17	Cite each included study and present its characteristics.	Page 4-11
Risk of bias in studies	18	Present assessments of risk of bias for each included study.	Page 5, 12, 13

Section and Topic	Item #	Checklist item	Location where item is reported
Results of individual studies	19	For all outcomes, present, for each study: (a) summary statistics for each group (where appropriate) and (b) an effect estimate and its precision (e.g. confidence/credible interval), ideally using structured tables or plots.	Page 6-20; supplementary information
Results of syntheses	20a	For each synthesis, briefly summarise the characteristics and risk of bias among contributing studies.	Page 6-20
	20b	Present results of all statistical syntheses conducted. If meta-analysis was done, present for each the summary estimate and its precision (e.g. confidence/credible interval) and measures of statistical heterogeneity. If comparing groups, describe the direction of the effect.	Page 6-20
	20c	Present results of all investigations of possible causes of heterogeneity among study results.	Page 20-22
	20d	Present results of all sensitivity analyses conducted to assess the robustness of the synthesized results.	Page 20-22
Reporting biases	21	Present assessments of risk of bias due to missing results (arising from reporting biases) for each synthesis assessed.	Page 20-22.
Certainty of evidence	22	Present assessments of certainty (or confidence) in the body of evidence for each outcome assessed.	Page 20-22
DISCUSSION			
Discussion	23a	Provide a general interpretation of the results in the context of other evidence.	Page 20-22
	23b	Discuss any limitations of the evidence included in the review.	Page 20-22
	23c	Discuss any limitations of the review processes used.	Page 20-22
	23d	Discuss implications of the results for practice, policy, and future research.	Page 20-22
OTHER INFORMATION			
Registration and protocol	24a	Provide registration information for the review, including register name and registration number, or state that the review was not registered.	N.A.
	24b	Indicate where the review protocol can be accessed, or state that a protocol was not prepared.	N.A.
	24c	Describe and explain any amendments to information provided at registration or in the protocol.	N.A.
Support	25	Describe sources of financial or non-financial support for the review, and the role of the funders or sponsors in the review.	Page 22
Competing interests	26	Declare any competing interests of review authors.	Page 22
Availability of data, code and other materials	27	Report which of the following are publicly available and where they can be found: template data collection forms; data extracted from included studies; data used for all analyses; analytic code; any other materials used in the review.	N.A.

Supplementary table S2. Supplementary study characteristics

Author (year)	Age (years)	BMI (kg/m²)	First-line n (%)<br>Second-line or higher n (%)	Level of CT	Software image analysis	Timing of baseline scan	Concomitant treatment
Akce (2021)[19]	Median 66	26 (46%) <25<br>31 (54%) ≥25	16 (28%)<br>41 (72%)	L3	SliceOmatic	Within 2 months before start of immunotherapy	2 (4%) received unspecified concomitant treatment
Antoun (2023)[20]	-	-	-	L3	SliceOmatic	Within 30 days before start of immunotherapy	6 (<1%) chemotherapy
Arribas (2021)[21]	Mean 57.7	Mean 23.8	22 (36%)<br>39 (64%)	L3	SliceOmatic	Within 10 days before start of immunotherapy	41 (67%) received unspecified concomitant treatment
Ashton (2023)[22]	Median 66	Mean 23.79	39 (20%)<br>161 (80%)	L3	ImageJ	Before start of immunotherapy	-
Aslan (2022)[23]	30 (58%) <65<br>22 (42%) ≥65	26 (50%) <25<br>15 (29%) 25-29.9<br>11 (21%) ≥30	All second-line or beyond	L3	ImageJ	Within 1 month before start of immunotherapy	-
Baldessari (2021)[24]	Median 70	Median 23.5	All first-line	L3	GE advanced workstation	Before start of immunotherapy	16 (36%) radiotherapy
Chen (2023)[25]	Mean 59.5	Mean 23.7	65 (47%)<br>73 (53%)	L3	3D slicer	Within 1 month before start of immunotherapy	63 (46%) anti-angiogenic therapy
Cortellini (2020)[26]	Median 66	Median 25	30 (30%)<br>70 (70%)	L3	Osirix lite	Within 3 months before start of immunotherapy	-
Crombe (2020)[27]	Median 63	Median 23.78	16 (14%)<br>101 (86%)	L3	SliceOmatic	Within 28 days before start of immunotherapy	-
Deng (2024a)[28]	-	-	69 (56%)<br>55 (44%)	L3	3D slicer	Before start of immunotherapy	50 (40%) SOX<br>38 (31%) XELOX<br>36 (29%) other chemotherapy
Deng (2024b)[29]	Mean<br>Sarcopenic 57.44<br>Non-sarcopenic 57.40	Mean<br>Sarcopenic 19.88<br>Non-sarcopenic 22.75	-	L3	3D slicer	-	115 (100%) received unspecified concomitant treatment
Fang (2024)[30]	Mean 63.1	Mean 23.0	93 (70%)<br>40 (30%)	L3	3D slicer	Within 2 months before start of immunotherapy<br>Median 8 days	-

Faron (2021)[31]	Mean 62	Mean 27	-	L3-L4	Automated deep-learning model	At treatment start	-
Feng (2024)[32]	Median 67	12 (17%) <18.5<br>46 (66%) 18.5-24.9<br>12 (17%) ≥25	55 (79%)<br>15 (21%)	L3	3D slicer	Within 1 month before start of immunotherapy	59 (84%) CRT<br>10 (14%) anti-VEGF
Fukata (2022)[33]	Median 70	-	All second-line or beyond	L3	-	Within 30 days before start of immunotherapy	No concomitant treatment
Fukushima (2020)[34]	Median 74	22 (79%) <25<br>6 (21%) ≥25	All second-line or beyond	L3	Synapse vincent	Within 1 month before start of immunotherapy	No concomitant treatment
Ged (2022)[35]	Median 63	60 (29%) <25<br>71 (35%) 25-30<br>74 (36%) ≥30	61 (30%)<br>144 (70%)	L3	SliceOmatic	Within 60 days before start of immunotherapy	-
Guo (2022)[36]	-	-	-	L3	Phillips Intelli Space Portal	Before start of immunotherapy	64 (66%) TKI
Haik (2021)[37]	Mean 61.9	Mean 23.8	46 (18%)<br>215 (82%)	L3	MEDASYS	Within 4 weeks before start of immunotherapy	Unspecified numbers concomitant anti-angiogenic therapy or chemotherapy
Imai (2025)[38]	Median 73	-	19 (9%)<br>195 (91%)	L3	Synapse vincent	-	-
Ishihara (2024)[39]	68 (43%) ≤65<br>91 (57%) >65	Median 23.1	All first-line	L3	Synapse vincent	Within 2 months before start of immunotherapy	75 (47%) TKI
Khan (2023)[40]	Mean 67.5	Mean 26.1	24 (25%)<br>73 (75%)	L3	SliceOmatic	Median 20 days before start of immunotherapy	-
Kim (2021a)[41]	Median 61.3	Median 22.85	-	L3	MATLAB	Before start of immunotherapy	10 (14%) radiotherapy
Kim (2021b)[42]	Mean 57.0	Mean 21.1	All second-line or beyond	L3	BMI measurements tools	Within 3 months before start of immunotherapy<br>Median 6.6 days	No concomitant treatment
Lee (2023)[43]	Mean 64.3	Mean men 23.0<br>women 22.6	153 (19%)<br>667 (81%)	L3	AID-U	Within 90 days before start of immunotherapy	-
Lyu (2023)[44]	Mean<br>Sarcopenic 59.09<br>Non-sarcopenic 59.08	Mean<br>Sarcopenic 22.12 Non-sarcopenic 22.91	All first-line	L3	SliceOmatic	Within 4 weeks before start of immunotherapy	56 (43%) chemotherapy

Magri (2019)[45]	Median 65	Mean 24.56	All second-line or beyond	L3	-	Within 10 weeks before or 2 months after start of immunotherapy	-
Makrakis (2023)[46]	Median 68	Mean 26.67	9 (17%)<br>43 (83%)	L3	SliceOmatic	Before start of immunotherapy	2 (4%) chemotherapy
Matsumoto (2022)[47]	Median 77	-	All first-line	L3	SliceOmatic	Within 6 weeks before start of immunotherapy	No concomitant treatment
Mcmanus (2023)[48]	Median 62	Median 27.3	All first-line	L3	ABACS	Within 6 weeks before start of immunotherapy<br>Median 22 days	No concomitant treatment
Mengoni (2024)[49]	Median 63	-	All first-line	L3	ImageJ	Within 120 days before start of immunotherapy	-
Roch (2020)[50]	Mean 63.54	18 (13%) <18.5<br>76 (54%) 18.5-24.9<br>38 (27%) 25-29.9<br>10 (7%) >30	19 (13%)<br>123 (87%)	L3	AW Volume Share 7	Before start of immunotherapy	-
Takada (2020)[51]	Median 67	Median men 21.9<br>women 19.8	17 (17%)<br>86 (83%)	L3	OsiriX lite	Before start of immunotherapy	-
Takei (2023)[52]	Median 71	Median 22.9	All first-line	L3	Synapse vincent	Within 30 days before start of immunotherapy	26 (43%) TKI
Takenaka (2022)[53]	Median 65	Median 19.39	50 (44%)<br>65 (56%)	L3	BMI_CT_ver6	Within 6 months before start of immunotherapy	-
Toshida[a] (2022)[54]	Median<br>Sarcopenic 72<br>Non-sarcopenic 71	Median<br>Sarcopenic 22.7<br>Non-sarcopenic 23.9	15 (43%)<br>20 (57%)	L3	-	Within 1 month before start of immunotherapy	-
Trestini (2024)[55]	Median 69	Median 24.5	All first-line	L3	SliceOmatic	Within 3 months before start of immunotherapy	55 (41%) radiotherapy
Ucgul (2024)[56]	57 (57%) ≥60	Median<br>Sarcopenic men 23.6; women 24.5<br>Non-sarcopenic men 28.0; women 26.8	-	L3	-	Within 6 months before start of immunotherapy	-
Ueki (2022)[57]	Sarcopenic<br>47 (66%) <75<br>24 (34%) ≥75	Sarcopenic<br>56 (79%) < 25<br>15 (21%) ≥25	All second-line or beyond	L3	OsiriX	At treatment start	-

	Non-sarcopenic<br>18 (72%) <75<br>7 (28%) ≥75	Non-sarcopenic<br>16 (64%) <25<br>9 (36%) ≥25					
Uojima (2023)[58]	Median 72	Median 23.5	-	L3	SliceOmatic	Within 3 months before start of immunotherapy	-
Wang (2021)[59]	Median 55	84 (80%) < 25<br>21 (20%) ≥25	All second-line or beyond	L3	OsiriX	-	No concomitant treatment
Wang (2025)[60]	Mean 66.4	Mean 21.8	-	T5	Siemens Syngo.via	Within 1 month before start of immunotherapy	156 (80%) received unspecified concomitant treatment
Willemsen (2023)[61]	Mean 63.2	Mean 22.2	40 (41%)<br>58 (59%)	L3	SliceOmatic	Within 30 days before start of immunotherapy	No concomitant treatment
Xiao (2022)[62]	Mean 51.4	Median 22.2	18 (10%)<br>154 (90%)	L3	SliceOmatic	Within 1 month before start of immunotherapy	-
Xiong (2023)[63]	Median 55.5	Median 22.3	-	L3	QCT Pro work-station	Within 1 month before start of immunotherapy	-
Yang (2024)[64]	Mean 58.36	Mean 22.75	-	L3	Core Slicer	Within 1 month before start of immunotherapy	75 (78%) chemotherapy and TKI<br>21 (22%) CRT and TKI
Ying (2024)[65]	Mean 74.9	Mean 20.7	-	L3	ImageJ	Within 1 month before start of immunotherapy	-
Young (2020)[66]	Mean 61	Mean 29.2	134 (47%)<br>153 (53%)	L3	SliceOmatic and ABACS	Within 6 months before start of immunotherapy	-

[a]Toshida (2022) included 98 patients of which 35 were treated with immunotherapy. All other patients were treated with lenvatinib. Results were reported separately. therefore, this review only includes the subset treated with immunotherapy.

CRT = chemoradiotherapy; L3 = third lumbar vertebra; L4 = fourth lumbar vertebra; NSCLC = non-small cell lung carcinoma; SOX = S-1 and oxaliplatin; T5 = fifth thoracic vertebra; TKI = tyrosine kinase inhibitor; VEGF = vascular endothelial growth factor; XELOX = capecitabine and oxaliplatin

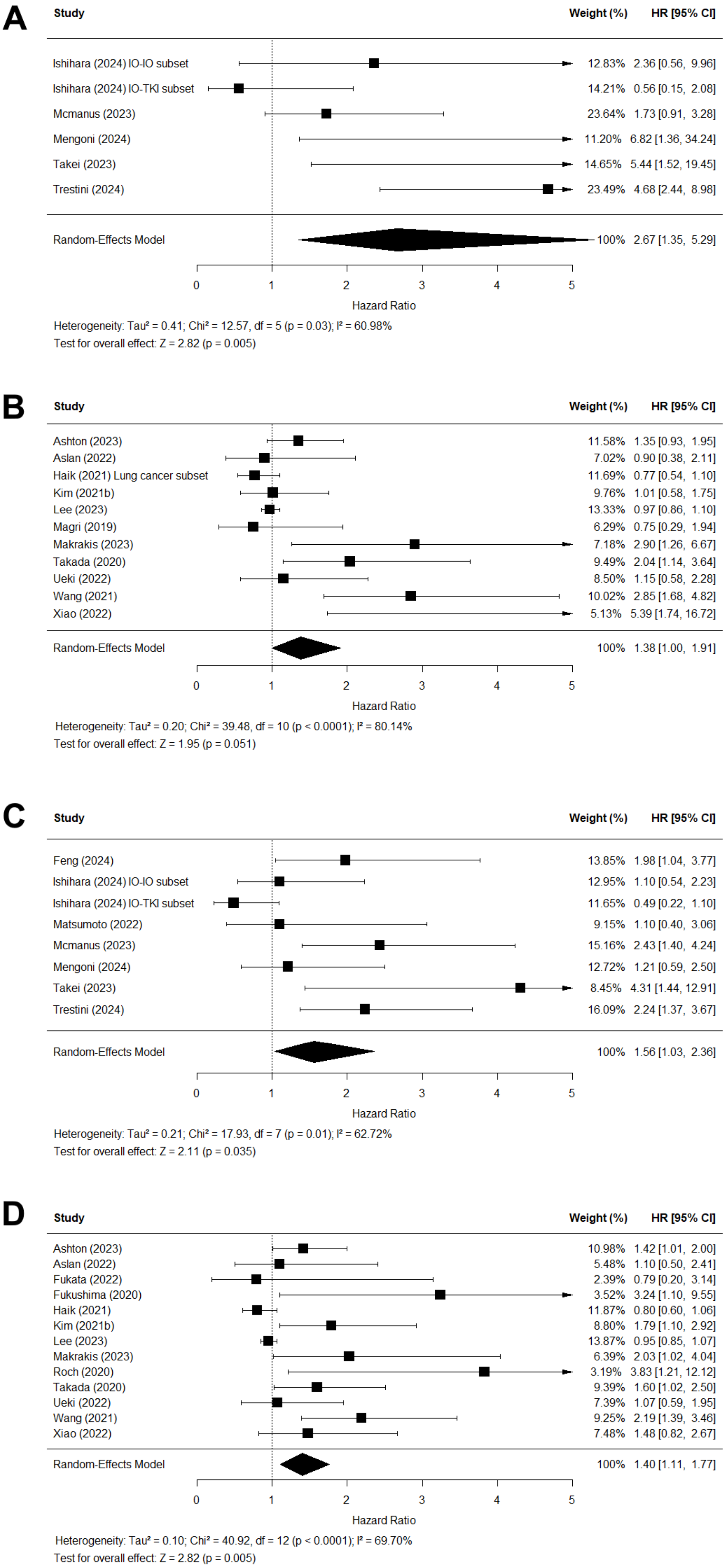
A
Study
Weight (%)
HR [95% CI]
Ishihara (2024) IO-IO subset 12.83% 2.36 [0.56, 9.96]
Ishihara (2024) IO-TKI subset 14.21% 0.56 [0.15, 2.08]
Mcmanus (2023) 23.64% 1.73 [0.91, 3.28]
Mengoni (2024) 11.20% 6.82 [1.36, 34.24]
Takei (2023) 14.65% 5.44 [1.52, 19.45]
Trestini (2024) 23.49% 4.68 [2.44, 8.98]
Random-Effects Model 100% 2.67 [1.35, 5.29]
0 1 2 3 4 5
Hazard Ratio
Heterogeneity: Tau² = 0.41; Chi² = 12.57, df = 5 (p = 0.03); I² = 60.98%
Test for overall effect: Z = 2.82 (p = 0.005)
B
Study
Weight (%)
HR [95% CI]
Ashton (2023) 11.58% 1.35 [0.93, 1.95]
Aslan (2022) 7.02% 0.90 [0.38, 2.11]
Haik (2021) Lung cancer subset 11.69% 0.77 [0.54, 1.10]
Kim (2021b) 9.76% 1.01 [0.58, 1.75]
Lee (2023) 13.33% 0.97 [0.86, 1.10]
Magri (2019) 6.29% 0.75 [0.29, 1.94]
Makrakis (2023) 7.18% 2.90 [1.26, 6.67]
Takada (2020) 9.49% 2.04 [1.14, 3.64]
Ueki (2022) 8.50% 1.15 [0.58, 2.28]
Wang (2021) 10.02% 2.85 [1.68, 4.82]
Xiao (2022) 5.13% 5.39 [1.74, 16.72]
Random-Effects Model 100% 1.38 [1.00, 1.91]
0 1 2 3 4 5
Hazard Ratio
Heterogeneity: Tau² = 0.20; Chi² = 39.48, df = 10 (p < 0.0001); I² = 80.14%
Test for overall effect: Z = 1.95 (p = 0.051)
C
Study
Weight (%)
HR [95% CI]
Feng (2024) 13.85% 1.98 [1.04, 3.77]
Ishihara (2024) IO-IO subset 12.95% 1.10 [0.54, 2.23]
Ishihara (2024) IO-TKI subset 11.65% 0.49 [0.22, 1.10]
Matsumoto (2022) 9.15% 1.10 [0.40, 3.06]
Mcmanus (2023) 15.16% 2.43 [1.40, 4.24]
Mengoni (2024) 12.72% 1.21 [0.59, 2.50]
Takei (2023) 8.45% 4.31 [1.44, 12.91]
Trestini (2024) 16.09% 2.24 [1.37, 3.67]
Random-Effects Model 100% 1.56 [1.03, 2.36]
0 1 2 3 4 5
Hazard Ratio
Heterogeneity: Tau² = 0.21; Chi² = 17.93, df = 7 (p = 0.01); I² = 62.72%
Test for overall effect: Z = 2.11 (p = 0.035)
D
Study
Weight (%)
HR [95% CI]
Ashton (2023) 10.98% 1.42 [1.01, 2.00]
Aslan (2022) 5.48% 1.10 [0.50, 2.41]
Fukata (2022) 2.39% 0.79 [0.20, 3.14]
Fukushima (2020) 3.52% 3.24 [1.10, 9.55]
Haik (2021) 11.87% 0.80 [0.60, 1.06]
Kim (2021b) 8.80% 1.79 [1.10, 2.92]
Lee (2023) 13.87% 0.95 [0.85, 1.07]
Makrakis (2023) 6.39% 2.03 [1.02, 4.04]
Roch (2020) 3.19% 3.83 [1.21, 12.12]
Takada (2020) 9.39% 1.60 [1.02, 2.50]
Ueki (2022) 7.39% 1.07 [0.59, 1.95]
Wang (2021) 9.25% 2.19 [1.39, 3.46]
Xiao (2022) 7.48% 1.48 [0.82, 2.67]
Random-Effects Model 100% 1.40 [1.11, 1.77]
0 1 2 3 4 5
Hazard Ratio
Heterogeneity: Tau² = 0.10; Chi² = 40.92, df = 12 (p < 0.0001); I² = 69.70%
Test for overall effect: Z = 2.82 (p = 0.005)

Supplementary Figure S1. Forest plots showing the subanalyses of the hazard ratios (HR) predicting the link between low skeletal muscle mass and survival stratified by first-line treatment and second-line or beyond. Studies were included in the subanalyses if 90% of their patients received immunotherapy as the specified treatment line. The combined effect of the studies is plotted with a black diamond. (A) The forest plot of the HR for overall survival (OS) in the subanalysis of studies with immunotherapy as first-line treatment. Ishihara (2024) performed separate analysis for their subset treated with dual immunotherapy (IO-IO) and the subset treated with immunotherapy and TKI (IO-TKI). (B) The forest plot of the HR for overall survival (OS) in the subanalysis of studies with immunotherapy as second-line or beyond. Haik (2021) performed PFS analysis on their entire population as well as the lung cancer subset. This plot shows the data for the lung subset. (C) The forest plot of the HR for progression-free survival (PFS) in the subanalysis of studies with immunotherapy as first-line treatment. Ishihara (2024) performed separate analysis for their subset treated with dual immunotherapy (IO-IO) and the subset treated with immunotherapy and TKI (IO-TKI). (D) The forest plot of the HR for progression-free survival (PFS) in the subanalysis of studies with immunotherapy as second-line or beyond.
